# Knowledge about infections is associated with antibiotic use: cross-sectional evidence from the health survey Northern Ireland

**DOI:** 10.1186/s12889-021-11018-x

**Published:** 2021-06-02

**Authors:** J. Shebehe, E. Ottertun, K. Carlén, D. Gustafson

**Affiliations:** 1 Clinical Epidemiology and Biostatistics, School of Medical Sciences, Örebro University, Örebro, Sweden; 2 School of Health Sciences, University of Skövde, Skövde, Sweden; 3 Department of Neurology, State University of New York Downstate Health Sciences University, Brooklyn, NY USA

**Keywords:** Health knowledge, attitudes, practice, Health literacy, Antibiotic resistance, Health education, Antibiotics

## Abstract

**Background:**

Antibiotic overuse is the main modifiable driver of antibiotic resistance. Factors associated with overuse have been inconsistently reported and vary across populations. Given the burgeoning occurrence of infectious diseases around the world, there remains a great need to identify barriers and solutions to the control of infections. We examined whether knowledge about infections and antibiotic resistance is associated with antibiotic use in a northern European population sample.

**Methods:**

The Health Survey Northern Ireland 2014/15 was completed by a cross-sectional sample of 4135 participants aged > 16 years. Participants were asked whether they had taken an antibiotic in the past 12 months; and six questions were asked concerning knowledge about infections and antibiotic resistance. Correct answers to the six knowledge questions defined a knowledge score (score range 0–6 correct answers). We used multivariable logistic regression to estimate odds of self-reported antibiotic use during the last 12 months in association with knowledge score (lowest score, 0/6, as referent), and response to each knowledge question. Covariates included sex, age group, smoking, alcohol drinking, deprivation index, self-rated health, and satisfaction with life. Results were outputted as Odds Ratios (OR) and 95% Confidence Intervals (CI).

**Results:**

Antibiotic use in the past 12 months was reported by 39.0% (1614/4135); and 84.2% (3482/4135) scored < 6/6 correct on knowledge statements. Compared to the lowest knowledge score (0/6 correct), the highest knowledge score (6/6 correct) was associated with higher odds of antibiotic use (adjusted OR 2.03, 95% CI [1.46, 2.81], *p* < 0.001), with a *P-*value < 0.001 for trend with increasing knowledge score. Female sex, age, high deprivation, and poor general health, were independently associated with higher odds of antibiotic use. Stratified analyses showed sex and age group differences.

**Conclusion:**

Knowledge, and other modifiable and non-modifiable risk factors, were positively associated with antibiotic use in the past 12 months. While the causal direction of these associations could not be determined, given the high prevalence of lesser knowledge, as well as independent contributions of other factors including socioeconomic characteristics, health literacy campaigns to raise awareness of antibiotic resistance should take a multi-pronged approach.

**Supplementary Information:**

The online version contains supplementary material available at 10.1186/s12889-021-11018-x.

## Background

Antimicrobial resistance (AMR) is a global crisis with serious consequences for the individual, public health, and the economy [1]. It has been estimated that during 2015, sixteen antibiotic resistant bacteria caused > 33,000 deaths and > 800,000 disability-adjusted life years (DALYs) in the European Economic Area [2]. By 2050, AMR is expected to cause > 1.3 million deaths in Europe and > 10 million deaths in the world annually [3]; low income countries and communities with health disparities are most affected [1, 4]. Recognition of the economic and health consequences of AMR has sparked global efforts to reduce the overuse of antibiotics.

Global commitment and multi-sector partnerships are critical to implement effective interventions to reduce unnecessary use of antibiotics and control AMR [5, 6]. Global survey data suggest that most antibiotics are used in non-hospitalized populations [5]. In the United Kingdom (UK) from 2013 to 2017, only 20% of antibiotics were prescribed in hospitals, whereas 80% were prescribed in outpatient settings, with no clinical justification for at least 30% [7, 8]. Despite this, in-hospital interventions have predominated and preventive interventions in the general public or community have been overlooked [9–11].

The World Health Organization’s (WHO) global action plan on AMR has five objectives, the first of which is to improve knowledge and understanding of AMR through communication, education and training [12]. National initiatives have adopted this plan, including the UK’s 20-year vision to combat AMR by 2040. The UK’s vision has nine goals, one of which is to make sure local communities understand all aspects of AMR [13]. Underscoring both WHO and UK missions, is that knowledge promotes appropriate antibiotic use and reduces antibiotic overuse and AMR. However, the association between knowledge and antibiotic use in the general population remains unclear [14].

One model targeting behavioural change related to AMR suggests that knowledge, environmental change, social/professional roles, social influences, and public beliefs and priorities, among others, are important factors. Thus, multipronged public health interventions are necessary to address AMR and to foster behaviour change [15]. Qualitative studies indicate that the public is unaware of their role and responsibility in combatting AMR [16]. The public needs to be engaged via simple, awareness-raising messages [17]. At the same time, published quantitative data are conflicting. For example, some studies report that people with low knowledge about antibiotics are more likely to use antibiotics [18]. Others report that people with more knowledge about antibiotics are more likely to use antibiotics [19, 20]; and others show no association [21, 22]. To inform public health interventions, it remains critical to crystallize how knowledge is associated with the likelihood of antibiotic use at the population level.

Given this backdrop, we took advantage of a big data resource – the Health Survey Northern Ireland 2014/15 (HSNI14/15) – to examine the association between knowledge regarding infections and AMR and antibiotic use. Since multi-prong interventions are necessary to decrease AMR, HSNI data allowed us to consider sex, age group, sociodemographic and behavioural factors, deprivation, and self-rated general health and satisfaction with life. These factors have been previously associated with knowledge and antibiotic use [23–27]. Given the conflicting evidence base about the temporality and direction of the relationship between knowledge and behaviour, our non-directional hypothesis was that the exposure, knowledge of treatment of common infectious diseases and AMR would be associated with the outcome, self-reported antibiotic use in the past 12 months.

## Methods

### Participants: health survey Northern Ireland 2014/15

The HSNI is a series of independent, cross-sectional surveys conducted annually since 2010 by Northern Ireland Statistics Research on behalf of the Northern Ireland Department of Health, Social Services and Public Safety. The HSNI examines a wide range of public health issues that are important for the people of Northern Ireland. In this analysis, we utilised data from HSNI14/15, conducted between April 1, 2014 and March 31, 2015. HSNI14/15 consisted of a random sample of 5850 street addresses that were representative of the general population in Northern Ireland [28]. A vacant address was not eligible for inclusion.

Eligible households were sent a letter of invitation via post. The letter stated that the survey was being conducted on behalf of the Department of Health, Social Services and Public Safety, Central Survey Unit, which is the leading social survey research organisation in Northern Ireland. The letter also stated that an official interviewer would visit the household to explain more about the study and that the survey could be rescheduled if necessary. Non-response occurred when an eligible address was not reached by an interviewer or when respondents refused to participate. To minimise non-response, an interviewer attempted up to four calls to each eligible household unless the respondents had previously refused to participate.

HSNI14/15 surveyed knowledge of infections, antibiotics and antibiotic resistance, and use of antibiotics over the past 12 months. Additionally, the survey collected sociodemographic, behavioural (smoking and alcohol drinking habits), and general health factors information [28].

### Ethical approval

The study protocol of HSNI14/15 was approved by Northern Ireland Department of Health. Informed consent was provided when the interviewer visited the household. Participants were able to refuse to participate at any stage of the survey [29]. No incentive was provided for participation. Data analyses presented in this paper were solely a secondary analysis of publicly available HSNI data. Administrative permission was necessary to access and use these HSNI data. Administrators of HSNI granted this access, provided the data file, and approved their use to the first author, JS.

### Data collection

Survey data from HSNI14/15 were collected using Computer-Assisted Personal Interviewing (CAPI). CAPI is a face-to-face interviewing method. HSNI14/15 interviewers visited the respondents with a computer, read the questions to the respondents, and entered the responses immediately on the computer during the course of the interview. If a participant did not want to engage in a CAPI interview, they were offered a self-administered, closed booklet version of the HSNI14/15 that contained the questions and response options. Responses were manually entered later [28].

### Study variables

Our primary exposure and outcome variables were located in the ‘Medication’ section of the HSNI14/15. Within this section, there was a subsection dedicated to antibiotics. This section was comprised of seven questions.

#### Primary outcome variable: antibiotic use

The answer to the first question in the ‘Medications’ section, subsection ‘Antibiotics’, was the primary outcome used in our analysis. The first question was ‘In the past 12 months, have you taken an antibiotic?’ Response options included: ‘Yes’, ‘No’ or ‘Don’t know’ [28].

#### Primary exposure variables: knowledge about infections, antibiotics and antibiotic resistance

The following six questions in the section on ‘Medications’, subsection ‘Antibiotics’, were used as the primary exposure. These six questions assessed knowledge about infections, antibiotics and antibiotic resistance (Table 1). Each question had the response options: ‘true’, ‘false’, or ‘don’t know’. The six statements and correct responses were as follows: 1) Colds and flus should be treated with antibiotics (false). 2) Antibiotics are used to treat bacterial infections (true). 3) Once you start to feel better, you should stop taking the antibiotic (false). 4) You increase your chances of developing drug-resistant bacteria if you take antibiotics when you don’t need to (true). 5) You increase your chances of developing drug-resistant bacteria if you do not finish the course of antibiotics (true). 6) Diseases such as tuberculosis, pneumonia and meningitis are becoming more difficult to treat, as drug-resistant bacteria do not respond to antibiotics and continue to cause infection (true) [28].

**Table 1:** Number and proportion of participants correctly responding to statements regarding knowledge about infections, antibiotics and antibiotic resistance

	Knowledge about infections, antibiotics, and antibiotic resistance		
		***Correct response***	Correct response ***N*** (% of 4135)
1	Colds and flus should be treated with an antibiotic	***False***	3805 (92.0)
2	Antibiotics are used to treat bacteria	***True***	2687 (65.0)
3	Once you start to feel better, you should stop taking the antibiotic	***False***	3828 (92.6)
4	Chances of resistant bacteria increase if you take antibiotics when you don’t need to.	***True***	2297 (55.6)
5	Chances of resistant bacteria increase if you don’t finish the course.	***True***	1809 (43.7)
6	Diseases such as tuberculosis, pneumonia and meningitis are becoming more difficult to treat, as drug-resistant bacteria do not respond to antibiotics and continue to cause infection	***True***	2072 (50.1)

To examine how knowledge about infections, antibiotics and antibiotic resistance was associated with antibiotic use in the past 12 months, the knowledge variable was considered as a correct answer to each individual statement (0 = incorrect; 1 = correct), as well as a composite knowledge score. The knowledge score was the sum of answers to all six statements. The score range was 0–6, where a score of 0 corresponded to incorrect answers on all 6 statements and a score of 6 was a correct answer to all statements.

### Potential covariates and confounders

We selected potential covariates and confounders based on published associations with knowledge, behaviour, and awareness and antibiotic use [23], as well as availability in the HSNI data set.

*Demographic characteristics*: included sex (Male/Female) and age group (16–24, 25–44, 45–64, and > 65 years) [24].

*Health behaviours****:*** included current cigarette smoking and alcohol drinking [27]. Current cigarette smoking was an answer of ‘yes’ to the question, ‘Do you smoke cigarette at all nowadays?’. Current alcohol drinking was an answer of ‘yes’ to the question, ‘Do you ever drink alcohol nowadays, including drinks you brew or make at home?’

*Sociodemographic factors****:*** included education attained and income [25]. A deprivation index derived from the Northern Ireland Multiple Deprivation Measure 2010 (NIMDM) [30] was used to estimate socioeconomic and sociodemographic disparities. The NIMDM combines geographical rankings of seven domains of deprivation weighted as follows: income (25%), employment (25%), health deprivation and disability (15%), education, skills and training (15%), proximity to services (10%), living environment (5%), and crime and disorder (5%). Quintiles of NIMDM rankings based on a participant’s home address comprise the deprivation index. Quintile 1 constitutes the most deprived whereas quintile 5 is the least deprived.

*General health status:* Self-rated general health and life satisfaction were components of the HSNI [26]. Self-rated general health over the past twelve months was described as ‘good’, ‘fairly good’ and ‘not good’. Life satisfaction was rated as ‘very satisfied’, ‘satisfied’, ‘neither satisfied nor dissatisfied’, ‘dissatisfied’, and ‘very dissatisfied’. We transformed the five life satisfaction categories into three: satisfied (including very satisfied and satisfied), neither satisfied nor dissatisfied, and dissatisfied (including dissatisfied and very dissatisfied).

### Statistical analyses

Participant characteristics and knowledge about infections and antibiotic resistance were described using frequencies and percentages. The Pearson’s chi-squared test was used to test for associations between categorical variables.

To examine the association between the exposure, knowledge about infections and antibiotic resistance and the outcome, self-reported antibiotic use over the past 12 months, weighted logistic regression analyses were used with ‘no reported use’ as the referent. Sampling weights were used to estimate robust standard errors in both unadjusted and adjusted models. The HSNI14/15 sample was weighted by sex and age using the Northern Ireland 2014 mid-year population estimates. Odds ratios (OR) and 95% confidence intervals (CI) were estimated.

The primary exposures were: 1) a correct answer to each of six knowledge statements, and 2) a composite knowledge score derived as the sum of correct answers to the six knowledge statements about infections and antibiotic resistance. Knowledge scores range from 0 to 6 with a score of 0 representing 0 correct answers to all 6 statements, and a score of 6 representing 6 correct answers to all 6 statements. First, we ran six models estimating whether the correct answer to each individual statement was associated with antibiotic use in the past 12 months. Second, using the sum of correct answers to the six statements regarding knowledge about infections and antibiotic resistance, we estimated whether knowledge score (maximum score 6/6 correct answers) was associated with antibiotic use in the past 12 months when compared to a lower knowledge score (score < 6/6 correct answers).

Univariate logistic regression analyses were followed by multivariable analyses. Sex, age group, smoking and alcohol drinking, deprivation index, self-rated general health, and satisfaction with life, were evaluated a priori as potential covariates in the multivariable models. Potential covariates with a *p* < 0.10 in univariate logistic analyses were included in multivariable models. Multivariable models were later stratified by sex and age group (removing sex and age as covariates, respectively). Results were considered statistically significant at *p* < 0.05 using two-sided tests. All statistical analyses were performed using Stata 16.0.

## Results

Of 5850 randomly selected street addresses, 981 were ineligible since the address was vacant, leaving an effective sample of 4869 households. Among these households, 28.6% (1394 of 4869 households) refused to participate and 8.1% (392 of 4869 households) were not reached. Thus, 63.3% (3083 of 4869) of households completed the survey, with some households providing data from more than one resident. As a result, HSNI14/15 data provided by the UK Data Service included 4207 individuals aged 16 years and older (1.36 individuals per household). Of these 4207 participants, 1.8% (*n* = 72) had missing data for questions comprising knowledge about infections and antibiotic resistance, self-reported antibiotic use, current cigarette smoking status, drinking status, self-rated general health, satisfaction with life or sample weight, and were excluded. This left 4135 participants included in our analysis. The majority of these HSNI14/15 participants were women (58.9%), older than 25 years (94.0%), current non-smokers (79.2%), and current alcohol drinkers (75.3%). There was a relatively equal distribution of participants across deprivation quintiles.

Among all 4135 participants, use of an antibiotic in the past 12 months was self-reported among 39.0% (*n* = 1614). Women were more likely to have used an antibiotic compared to men (64.7% vs 35.3%, *p* < 0.001). Participants who provided correct answers to the six statements regarding knowledge about infections and antibiotic resistance, which comprised our primary exposure, are presented in Table 1. Eight percent (*n* = 334) of participants responded ‘don’t know’ to all six questions.

HSNI14/15 participant characteristics by self-reported antibiotic use in the past 12 months are presented in Table 2. Notably self-reported antibiotic use was associated with all characteristics we considered including knowledge score, sex, age group, current smoking status, deprivation index, general health and satisfaction with life (*p*’s < 0.10), except alcohol drinking (*p* = 0.321). Therefore, alcohol drinking was not included in multivariable-adjusted models.

**Table 2:** Characteristics of 4135 HSNI14/15 participants by self-reported antibiotic use in the past 12 months

Characteristics	Use of antibiotics in the past 12 months			
	No *N* = 2521 *n* (%)	Yes *N* = 1614 *n* (%)	Chi-square^†^ (df)	*P*^†^
Knowledge about infections and antibiotic resistance			31.62 (6)	< 0.001
*0 (lowest knowledge)*	253 (10.0)	100 (6.2)		
*1*	70 (2.8)	45 (2.8)		
*2*	99 (3.9)	61 (3.8)		
*3*	517 (20.5)	374 (23.2)		
*4*	631 (25.0)	380 (23.5)		
*5*	592 (23.5)	360 (22.3)		
*6 (highest knowledge)*	359 (14.2)	294 (18.2)		
Sex			36.44 (1)	< 0.001
*Male*	1129 (44.8)	570 (35.3)		
*Female*	1392 (55.2)	1044 (64.7)		
Age group (years)			10.53 (3)	0.015
*16–24*	153 (6.1)	94 (5.9)		
*25–44*	793 (31.4)	503 (31.1)		
*45–64*	899 (35.6)	515 (31.9)		
*>* *65*	676 (26.9)	502 (31.1)		
Current smoker, Yes	485 (19.3)	376 (23.3)	9.83 (1)	0.002
Alcohol drinker, Yes	1920 (76.2)	1193 (73.9)	2.66 (1)	0.102
Deprivation quintiles			29.34 (4)	< 0.001
*1 (most deprived)*	362 (14.4)	306 (19.0)		
*2*	490 (19.4)	343 (21.2)		
*3*	536 (21.2)	363 (22.5)		
*4*	592 (23.5)	325 (20.2)		
*5 (least deprived)*	541 (21.5)	277 (17.1)		
General health			209.55 (2)	< 0.001
*Good*	1643 (65.2)	738 (45.7)		
*Fairly good*	610 (24.2)	458 (28.4)		
*Not good*	268 (10.6)	418 (25.9)		
Satisfaction with life			33.47 (2)	< 0.001
*Satisfied*	2258 (89.6)	1358 (84.1)		
*Neither satisfied nor dissatisfied*	177 (7.0)	144 (8.9)		
*Dissatisfied*	86 (3.4)	112 (7.0)		

†: Two-sided Pearson chi-square statistics (degrees of freedom) and *P* value

HSNI: Health Survey Northern Ireland

The association between knowledge of infections and antibiotic resistance and antibiotic use was estimated using weighted odds of antibiotic use in the past 12 months predicted by knowledge defined by a correct answer to each individual statement, as well as knowledge score (Table 3). Evaluation of individual statements showed a 61% higher odds (multivariable-adjusted OR 1.61, 95% CI [1.38,1.88], *p* < 0.001) of antibiotic use associated with a correct answer to the statement ‘Antibiotics are used to treat bacteria’ and 19% higher odds (adjusted OR 1.19, 95% CI [1.03,1.37], *p* < 0.001) of antibiotic use with a correct answer to the statement ‘Chances of resistant bacteria increase if you don’t finish the course’. A breakdown of knowledge score (the sum of correct answers to six statements about infections and antibiotic resistance (range 0–6)) by number correct answers showed that 8.5% (*N* = 353) of participants had 0/6 correct answers, 2.8% (*N* = 115) had 1/6 correct answers, 3.8% (*N* = 160) had 2/6 correct answers, 21.6% (*N* = 891) had 3/6 correct answers, 24.6% (*N* = 1011) had 4/6 correct answers, 23.0% (*N* = 952) had 5/6 correct answers and 15.8% (*N* = 653) had 6/6 correct answers.

**Table 3:** Odds of self-reported antibiotic use by correct answer to individual knowledge statements. HSNI14/15

			Odds of self-reported antibiotic use in the past 12 months			
	**Model‡**		**Unadjusted**		**Adjusted**†	
	Statements regarding knowledge about infections and antibiotic resistance		**OR (95% CI)**	***P***	**OR (95% CI)**	***P***
1	Colds and flus should be treated with an antibiotic	Incorrect	1.00 (ref)		1.00 (ref)	
		Correct	1.15 (0.95–1.40)	0.156	1.01 (0.76–1.33)	0.967
2	Antibiotics are used to treat bacteria	Incorrect	1.00 (ref)		1.00 (ref)	
		Correct	1.53 (1.32–1.78)	< 0.001	1.61 (1.38–1.88)	< 0.001
3	Once you start to feel better, you should stop taking the antibiotic	Incorrect	1.00 (ref)		1.00 (ref)	
		Correct	1.33 (1.08–1.63)	0.007	1.15 (0.87–1.53)	0.335
4	Chances of resistant bacteria increase if you take antibiotics when you don’t need to.	Incorrect	1.00 (ref)		1.00 (ref)	
		Correct	0.99 (0.86–1.14)	0.909	1.03 (0.89–1.20)	0.653
5	Chances of resistant bacteria increase if you don’t finish the course.	Incorrect	1.00 (ref)		1.00 (ref)	
		Correct	1.20 (1.04–1.38)	0.011	1.19 (1.03–1.37)	0.021
6	Diseases such as tuberculosis, pneumonia and meningitis are becoming more difficult to treat, as drug-resistant bacteria do not respond to antibiotics and continue to cause infection	Incorrect	1.00 (ref)		1.00 (ref)	
		Correct	0.95 (0.83–1.10)	0.514	1.03 (0.89–1.19)	0.728

‡ Model 1 to 6: Unadjusted and adjusted odds ratio (OR) and 95% confidence interval (95% CI) with Robust standards errors for use of antibiotics in the past 12 months associated with correct answers to the six statements concerning infections and antibiotic resistance

† Adjusted for sex, age group, cigarette smoking, deprivation quintiles, self-rated general health, and satisfaction with life

HSHI: Health Survey Northern Ireland

Estimates of the odds of antibiotic use in the past 12 months associated with our primary exposure, knowledge score and each characteristic, were accomplished using weighted multivariable-adjusted models and are presented in Table 4. Higher knowledge score was associated with higher odds of antibiotic use (*p* for trend < 0.001), with adjustment for sex, age, current smoking, deprivation quintiles, and self-rated general health and satisfaction with life. However, knowledge was not the only characteristic associated with higher odds of antibiotic use. Women compared to men (adjusted OR 1.54, 95% CI [1.32, 1.79], *p* < 0.001) and those reporting poor versus good general health (adjusted OR 3.53, 95% CI [2.84, 4.40], *p* < 0.001) were more likely to report using an antibiotic in the past 12 months. Those aged 45–64 years compared to those age 16–24 years (adjusted OR 0.71, 95% CI [0.52, 0.96], *p* = 0.024) and those comprising the 5th deprivation quintile (the least deprived) compared to the referent 3rd deprivation quintile (adjusted OR 0.73, 95% CI [0.58, 0.92], *p* = 0.007) were less likely to report antibiotic use.

**Table 4:** Odds of self-reported antibiotic use by knowledge about infections and antibiotic resistance, and covariates. HSNI14/15

	Odds of self-reported antibiotic use in the past 12 months			
	Unadjusted		Adjusted†	
	OR (95% CI) ‡	***P***	OR (95% CI) ‡	***P***^***a***^
**Knowledge score (0–6)**				
0 (lowest knowledge)	1.00 (ref)		1.00 (ref)	
1	1.20 (0.73–1.98)	0.476	1.15 (0.65–2.01)	0.633
2	1.24 (0.79–1.94)	0.358	1.19 (0.76–1.86)	0.452
3	1.57 (1.15–2.12)	0.004	1.60 (1.16–2.20)	0.004
4	1.29 (0.96–1.75)	0.094	1.35 (0.98–1.85)	0.064
5	1.37 (1.02–1.86)	0.039	1.46 (1.06–2.00)	0.020
6 (highest knowledge)	1.81 (1.32–2.47)	< 0.001	2.03 (1.46–2.81)	< 0.001
**Sex**				
*Male*	1.00 (ref)		1.00 (ref)	
*Female*	1.57 (1.36–1.82)	< 0.001	1.54 (1.32–1.79)	< 0.001
**Age (years)**				
*16–24*	1.00 (ref)		1.00 (ref)	
*25–44*	1.02 (0.77–1.37)	0.873	0.93 (0.69–1.25)	0.637
*45–64*	0.91 (0.69–1.22)	0.539	0.71 (0.52–0.96)	0.024
*65+*	1.20 (0.90–1.61)	0.211	0.85 (0.63–1.16)	0.305
**Current smoker**				
*No*	1.00 (ref)		1.00 (ref)	
*Yes*	1.29 (1.09–1.53)	0.003	1.12 (0.94–1.35)	0.212
**Alcohol drinker**†				
*Non-drinker*	1.00 (ref)			
*Drinker*	0.92 (0.78–1.08)	0.314		
**Deprivation quintiles**				
*1(most deprived)*	1.14 (0.91–1.43)	0.254	1.01 (0.80–1.28)	0.922
*2*	0.95 (0.77–1.18)	0.656	0.94 (0.75–1.17)	0.591
*3*	1.00 (ref)		1.00 (ref)	
*4*	0.73 (0.59–0.90)	0.003	0.76 (0.61–0.95)	0.014
*5(least deprived)*	0.70 (0.56–0.87)	0.001	0.73 (0.58–0.92)	0.007
**Self-rated health**				
*Good*	1.00 (ref)		1.00 (ref)	
*Fairly*	1.66 (1.41–1.96)	< 0.001	1.71 (1.43–2.04)	< 0.001
*Not good*	3.43 (2.84–4.14)	< 0.001	3.53 (2.84–4.40)	< 0.001
**Satisfaction with life**				
*Satisfied*	1.00 (ref)		1.00 (ref)	
*Neither satisfied nor dissatisfied*	1.35 (1.05–1.73)	0.018	0.81 (0.62–1.07)	0.132
*Dissatisfied*	2.26 (1.66–3.09)	< 0.001	1.21 (0.82–1.78)	0.335

‡ Odds ratio (OR) and 95% confidence interval (95% CI) with Robust standards errors

† Adjusted for sex, age, cigarette smoking, deprivation quintiles, self-rated general health, and satisfaction with life based on *P* values < 0.250 in univariate analyses

*a*: *P* value for trend of knowledge score was < 0.001

HSNI: The Health Survey of Northern Ireland

Sex and age group stratification illustrated some nuances to these data. Among both women and men, the highest knowledge score (6/6 vs. 0/6 correct answers) was associated with 2 times higher odds of antibiotic use in the past 12 months; and poor self-rated health was linked to higher odds of antibiotic use. However, deprivation index was a characteristic associated with antibiotic use among men only, and when compared to the middle deprivation quintile, men comprising the least deprivation quintiles exhibited 42% lower odds of antibiotic use (Supplemental Table S1). Age group was a characteristic associated with antibiotic use among women only and women aged 45–64 years reported lower odds of antibiotic use.

Age group stratification (Supplemental Table S2) evidenced that the positive association between antibiotic use and knowledge was weakest among 16–24 year-olds. Being a woman was positively associated with antibiotic use among those ages 25–64 years; smoking among those age 45–64 years; and poorer than good self-rated general health among those age 25 years and older.

## Discussion

Data from the HSNI14/15 evidenced that higher knowledge about antibiotic use in common infections and antibiotic resistance was associated with higher multivariable-adjusted odds of self-reported antibiotic use in the past 12 months. This was especially observed among women, those 25 years or older, and those with poorer self-rated general health. Those who were least deprived were less likely to report antibiotic use in the past 12 months.

Our results support previous reports that people who are more knowledgeable about antibiotics and antibiotic resistance are more likely to use antibiotics than people who are less knowledgeable [19, 20]. Another study conducted in the United Kingdom, found that adults with good knowledge about antibiotics had better attitudes towards antibiotic use, but not lower antibiotic consumption [21]. A higher likelihood of antibiotic use among people with high knowledge about infections and antibiotic resistance suggests that this knowledge may be insufficient to curb higher use of antibiotics [31]. Contrastingly, antibiotic use may also lead to better knowledge about antibiotics.

Knowledge is only one component of health literacy, which is ‘the degree to which individuals have the capacity to obtain, process, and understand basic health information and services needed to make appropriate health decisions’ [32]. Health literacy influences the complete spectrum of health behaviour and health behaviour change to offset unhealthy behaviour [32]. Therefore, knowledge may be insufficient to influence antibiotic overuse. Good, multi-faceted health literacy is required to make the critical decisions necessary to reduce antibiotic overuse [33]. As reported previously, people with high knowledge about antibiotics reported higher antibiotic use, whereas people with good health literacy used an antibiotic significantly less frequently compared with people with insufficient health literacy [19]. Our finding of a positive association between knowledge and antibiotic use supports this finding. Those who use antibiotics may gain knowledge from prescribers, pharmacists, or other sources of health information such as the internet [21].

Since most published studies are cross-sectional, the causal relationship between knowledge and antibiotic use cannot be determined. Well-delivered knowledge is empowering and a prerequisite of health literacy [34], and informed choice is not possible unless people have the relevant knowledge. Experiences from the UK’s Antibiotic Guardian campaign suggest awareness-raising messages focused to people with poor antibiotic resistance knowledge are likely to be effective [17]. Still, HSNI14/15 data cannot be temporally elucidated.

Among HSNI14/15 participants, men and women did not differ on knowledge about infections and antibiotic resistance; however, women were more likely to report antibiotic use than men. Sex differences in health-seeking behaviour are observed in other Western societies where women seek healthcare more frequently than men [35]. Whether this is related to knowledge is unclear. Several studies show that women have more frequent contact with healthcare providers over their life course, compared to men. This may be almost solely due to female reproductive factors such as pregnancy, birthing, and the menopause, as well as experience with antibiotics while caring for children and other family members [36–38]. Multiple contacts with healthcare providers over time are great opportunities for health education to reduce undesired habits including antibiotic overuse. However, due to clinical time constraints and bottom lines related to clinical revenues, physicians tend to prescribe antibiotics more often than they inform on antibiotic overuse and the danger of antibiotic resistance [31].

Compared to other age groups, younger (16–24 years) HSNI14/15 participants comprised the largest proportion (92%, 228/247) with lesser knowledge about infections and antibiotic resistance, as has been previously reported [24, 39]. Younger people have had fewer opportunities to interact with the healthcare system, and are less likely to have assumed responsibility for child or aging parent care. Thus, they may lack experience with antibiotics, and consequently have less knowledge about health issues including infectious diseases and antibiotics [24].

In HSNI14/15, there is some evidence of a gradient of association between deprivation and antibiotic use. Compared with the 3rd deprivation quintile, the odds of antibiotic use in the past 12 months were not different among the most deprived (3rd and 2nd deprivation quintiles), but were lower among the least deprived (4th and 5th deprivation quintiles). NIMDM deprivation index is a composite of income, employment, health deprivation and disability, education, skills and training, proximity to services, living environment, and crime and disorder, with consideration for home address. Many studies have shown that individuals comprising lower socioeconomic classes tend to be less knowledgeable about infections and antibiotic resistance compared with individuals in higher socioeconomic classes [40]. While we cannot address which component of the NIMDM deprivation index drives the observed association with antibiotic use, this index is weighted most heavily for income (25%) and employment (25%). Our data concur, such that lesser deprivation was associated with lower odds of antibiotic use. It has also been shown that individuals experiencing the most deprivation are more likely to have multiple chronic diseases and a greater risk for infectious diseases. This has been markedly evident during the corona virus disease 2019 (COVID-19) pandemic [41].

Poorer self-rated general health was also associated with higher odds of antibiotic use in HSNI14/15. This may have been observed for several reasons, including: 1) co-occurring physical disease or co-morbidities, some of which may increase the risk of a bacterial infection [42]; 2) psychological distress that may adversely influence coping with common infections [43]; and 3) a higher likelihood of reporting clinical illness with infection for which physicians are prescribing antibiotics more readily [44].

### Strengths and limitations

Our analysis has several strengths. The HSNI14/15 includes a representative, population-based sample of more than 4000 participants, across a wide age range. Self-reported data were collected using well-validated scales and state-of-the-art CAPI methods without reliance on population-based prescribing statistics; and the calculated deprivation index is comprehensive and informative. In addition, multivariable analyses allow evaluation of the association between knowledge and antibiotic use after controlling for potentially relevant confounders, lending to the robustness of our result. Given the relatively few studies in this field, these data are highly valuable and fill a gap in the literature. Despite these strengths, as with many large-scale population-based surveys, there are limitations. First, the HSNI uses a serial cross-sectional design comprised of independent samples. Despite the agreement of our results with other published studies, we cannot determine causality, i.e., whether knowledge influences behaviour (antibiotic use) or vice versa. Second, HSNI data are self-reported, thus, there is inherent recall bias. Regarding the face-to-face efficiency of CAPI, underreporting is more frequent in face-to-face interviewing of sensitive, stigmatized or unpopular behaviours [45]. This risk was reduced in HSNI14/15 because alternatives, such as Computer-Assisted Self-Interview (CASI) or pen-and-paper, were available. Third, as the HSNI is geographically narrow in scope, our findings may not be generalizable to other regions of Europe, other ‘Western’ countries, or globally. However, even in Low- or Middle-Income countries, socioeconomics, sex and gender expectations, antibiotic prescribing practices, self-medication, and medicine regulations, are related to knowledge and antibiotic use in a similar fashion. For example, and as mentioned, individuals from lower socioeconomic classes tend to be less knowledgeable about infections and antibiotic resistance compared to those in higher socioeconomic classes [40], which is mimicked by our observation using the NIMDM deprivation index. Furthermore, sex differences in the association between knowledge about infections and antibiotic resistance and antibiotic use are universal, regardless of country or context [18, 39]. Fourth, we are unable to address response bias using this dataset. Fifth, as with any large-scale survey, there is a limit to the number of questions that can be asked. For example, in the HSNI, information is lacking on self-medication via sharing of prescription antibiotics [46], the role of different healthcare providers in facilitating accrual of knowledge [47], and the important contribution of responsible healthcare providers’ prescribing practices. Finally, given the need for multi-pronged interventions specific to reducing AMR, surveys providing the basis for AMR interventions need to provide the distinct data necessary to accomplish that goal [15].

## Conclusion

HSNI14/15 data illustrate that there was a positive cross-sectional association between knowledge about infections and antibiotic resistance and antibiotic use. However, we also observed a number of common modifiable and non-modifiable risk factors associated with both self-reported antibiotic use and basic knowledge of treatment of common infectious diseases and antibiotic resistance. These factors represent potential ‘at risk’ groups and thus, allow the creation of targeted campaigns that are critical for increasing knowledge in general, supporting informed choices, and raising the public’s health literacy. In addition, future research should prospectively examine whether knowledge about infections, antibiotics and antibiotic resistance predicts quantity or frequency of antibiotic use; as well as identify optimal types of information and methods of information dissemination that reduce antibiotic overuse. It is of critical importance that future prospective studies include measures of knowledge and attitudes, as well as dedicated measures of health literacy.

## Supplementary Information

**Additional file 1.**

**Additional file 2.**

## Data Availability

The datasets analysed during the current study are available in the UK Data Service repository, https://beta.ukdataservice.ac.uk/datacatalogue/studies/study?id=8347.

## Abbreviations

AMR: Antimicrobial resistance
CAPI: Computer-Assisted Personal Interview
CASI: Computer-Assisted Self-Interview
CI: Confidence interval
COVID-19: Corona virus disease 2019
HSNI: Health Survey Northern Ireland
NIMDM: Northern Ireland Multiple Deprivation Measure
OR: Odds ratio
UK: United Kingdom
USA: United States of America
WHO: World Health Organization
