# Supplementary material for: Knowledge about infections is associated with antibiotic use: cross-sectional evidence from the health survey Northern Ireland
Source: BMC Public Health. 2021 Jun 2;21:1041. doi: 10.1186/s12889-021-11018-x (PMC8170648; doi:10.1186/s12889-021-11018-x)
Supplement: Supplementary file 1 — Additional file 1. [file 12889_2021_11018_MOESM1_ESM.docx]

**Supplemental Table S1. Odds of antibiotic use by knowledge about infections and antibiotic resistance, stratified by sex. HSNI14/15.**

|  |  | **Odds of self-reported antibiotic use in the past 12 months** | | | | |
| --- | --- | --- | --- | --- | --- | --- |
|  |  | **Women (*n*=2436)** |  |  | **Men**  **(*n*=1699)** |  |
|  |  | **OR (95% CI) ‡** | ***P^a^*** |  | **OR (95% CI) ‡** | ***P^b^*** |
| **Knowledge score (0-6)** | |  |  |  |  |  |
|  | *0 (lowest knowledge)* | 1.00 (ref) |  |  | 1.00 (ref) |  |
|  | *1* | 0.96 (0.41–2.24) | 0.925 |  | 1.43 (0.68–3.02) | 0.343 |
|  | *2* | 1.06 (0.57–1.94) | 0.860 |  | 1.41 (0.72–2.77) | 0.310 |
|  | *3* | 1.51 (0.99–2.29) | 0.056 |  | 1.77 (1.08–2.89) | 0.023 |
|  | *4* | 1.33 (0.88–2.02) | 0.174 |  | 1.38 (0.85–2.26) | 0.192 |
|  | *5* | 1.34 (0.88–2.03) | 0.168 |  | 1.75 (1.07–2.87) | 0.026 |
|  | *6 (highest knowledge)* | 2.06 (1.34–3.17) | 0.001 |  | 2.00 (1.20–3.34) | 0.008 |
| **Age (years)** | |  |  |  |  |  |
|  | *16-24* | 1.00 (ref) |  |  | 1.00 (ref) |  |
|  | *25-44* | 0.96 (0.66–1.39) | 0.831 |  | 0.83 (0.50–1.36) | 0.454 |
|  | *45-64* | 0.67 (0.46–0.97) | 0.036 |  | 0.74 (0.45–1.21) | 0.228 |
|  | *65+* | 0.72 (0.49–1.06) | 0.094 |  | 1.11 (0.68–1.82) | 0.682 |
| **Current smoker** | |  |  |  |  |  |
|  | *No* | 1.00 (ref) |  |  | 1.00 (ref) |  |
|  | *Yes* | 1.11 (0.88–1.41) | 0.365 |  | 1.10 (0.82–1.48) | 0.505 |
| **Deprivation quintiles** | |  |  |  |  |  |
|  | *1 (most deprived)* | 1.23 (0.92–1.65) | 0.170 |  | 0.73 (0.49–1.09) | 0.123 |
|  | *2* | 1.13 (0.86–1.50) | 0.379 |  | 0.69 (0.48–0.99) | 0.044 |
|  | *3* | 1.00 (ref) |  |  | 1.00 (ref) |  |
|  | *4* | 0.87 (0.66–1.15) | 0.333 |  | 0.60 (0.42–0.86) | 0.005 |
|  | *5 (least deprived)* | 0.84 (0.63–1.12) | 0.236 |  | 0.58 (0.40–0.84) | 0.004 |
| **Self-rated health** | |  |  |  |  |  |
|  | *Good* | 1.00 (ref) |  |  | 1.00 (ref) |  |
|  | *Fairly good* | 1.60 (1.29–2.00) | <0.001 |  | 1.89 (1.42–2.52) | <0.001 |
|  | *Not good* | 4.23 (3.18–5.62) | <0.001 |  | 2.66 (1.85–3.82) | <0.001 |
| **Satisfaction with life** | |  |  |  |  |  |
|  | *Satisfied* | 1.00 (ref) |  |  | 1.00 (ref) |  |
|  | *Neither satisfied nor dissatisfied* | 0.88 (0.61–1.26) | 0.471 |  | 0.71 (0.46–1.10) | 0.122 |
|  | *Dissatisfied* | 1.03 (0.64–1.65) | 0.895 |  | 1.58 (0.86–2.93) | 0.143 |

**‡** Adjusted odds ratio (OR) and 95% confidence interval (95% CI) with Robust standards error. Adjusted for age, cigarette smoking, deprivation quintiles, self-rated general health, and satisfaction with life.

*a*: *P* value for trend of knowledge score was 0.005 for women.

*b*: *P* value for trend of knowledge score was 0.023 for men.

HSNI: Health Survey of Northern Ireland.
