# Supplementary material for: Knowledge about infections is associated with antibiotic use: cross-sectional evidence from the health survey Northern Ireland
Source: BMC Public Health. 2021 Jun 2;21:1041. doi: 10.1186/s12889-021-11018-x (PMC8170648; doi:10.1186/s12889-021-11018-x)
Supplement: Supplementary file 2 — Additional file 2. [file 12889_2021_11018_MOESM2_ESM.docx]

**Supplemental Table S2. Odds of antibiotic use by knowledge about infections and antibiotic resistance, stratified by age-group. HSNI14/15.**

|  | | **Odds of self-reported antibiotic use in the past 12 months** | | | | | | | | | | |
| --- | --- | --- | --- | --- | --- | --- | --- | --- | --- | --- | --- | --- |
|  | | **Age 16-24 years (*n*=247)** | |  | **Age 25-44 years (*n*=1296)** | | | **Age 45-64 years (*n*=1414)** | |  | **Age 65+years (*n*=1178)** | |
|  |  | **OR (95% CI) ‡** | ***P^a^*** |  | **OR (95% CI) ‡** | ***P^b^*** |  | **OR (95% CI) ‡** | ***P^c^*** |  | **OR (95% CI) ‡** | ***P^d^*** |
| **Knowledge score (0-6)** | |  |  |  |  |  |  |  |  |  |  |  |
|  | *0 (lowest knowledge)* | 1.00 (ref) |  |  | 1.00 (ref) |  |  | 1.00 (ref) |  |  | 1.00 (ref) |  |
|  | *1* | 0.20 (0.02–1.62) | 0.132 |  | 1.06 (0.37–3.00) | 0.918 |  | 4.00 (1.42–11.23) | 0.009 |  | 1.66 (0.78–3.50) | 0.188 |
|  | *2* | 0.31 (0.09–1.09) | 0.069 |  | 1.12 (0.50–2.49) | 0.783 |  | 2.63 (1.14–6.08) | 0.023 |  | 1.36 (0.68–2.71) | 0.382 |
|  | *3* | 0.39 (0.14–1.11) | 0.078 |  | 1.97 (1.13–3.44) | 0.016 |  | 2.94 (1.63–5.28) | <0.001 |  | 1.83 (1.16–2.89) | 0.010 |
|  | *4* | 0.34 (0.13–0.93) | 0.036 |  | 1.79 (1.04–3.08) | 0.035 |  | 2.06 (1.15–3.66) | 0.014 |  | 1.72 (1.07–2.75) | 0.024 |
|  | *5* | 0.40 (0.13–1.24) | 0.113 |  | 1.66 (0.96–2.87) | 0.069 |  | 2.98 (1.68–5.29) | <0.001 |  | 1.35 (0.83–2.20) | 0.220 |
|  | *6 (highest knowledge)* | 0.39 (0.11–1.38) | 0.144 |  | 2.45 (1.37–4.39) | 0.003 |  | 3.87 (2.16–6.93) | <0.001 |  | 2.05 (1.24–3.41) | 0.005 |
| **Sex** | |  |  |  |  |  |  |  |  |  |  |  |
|  | *Male* | 1.00 (ref) |  |  | 1.00 (ref) |  |  | 1.00 (ref) |  |  | 1.00 (ref) |  |
|  | *Female* | 1.49 (0.81–2.74) | 0.204 |  | 1.83 (1.42–2.37) | <0.001 |  | 1.56 (1.23–1.99) | <0.001 |  | 1.12 (0.88–1.43) | 0.368 |
| **Current smoker** | |  |  |  |  |  |  |  |  |  |  |  |
| *No* | | 1.00 (ref) |  |  | 1.00 (ref) |  |  | 1.00 (ref) |  |  | 1.00 (ref) |  |
| *Yes* | | 1.33 (0.69–2.57) | 0.396 |  | 0.93 (0.70–1.24) | 0.626 |  | 1.34 (1.01–1.78) | 0.041 |  | 0.87 (0.60–1.27) | 0.474 |
| **Deprivation quintiles** | |  |  |  |  |  |  |  |  |  |  |  |
|  | *1 ((most deprived)* | 0.45 (0.19–1.07) | 0.072 |  | 1.21 (0.82–1.77) | 0.342 |  | 1.04 (0.70–1.55) | 0.842 |  | 1.21 (0.80–1.82) | 0.359 |
|  | *2* | 0.56 (0.25–1.24) | 0.152 |  | 1.06 (0.74–1.52) | 0.753 |  | 1.10 (0.76–1.59) | 0.610 |  | 0.91 (0.61–1.34) | 0.622 |
|  | *3* | 1.00 (ref) |  |  | 1.00 (ref) |  |  | 1.00 (ref) |  |  | 1.00 (ref) |  |
|  | *4* | 0.34 (0.14–0.82) | 0.017 |  | 0.83 (0.57–1.19) | 0.309 |  | 0.84 (0.58–1.20) | 0.340 |  | 0.90 (0.62–1.31) | 0.583 |
|  | *5 (least deprived)* | 0.50 (0.20–1.24) | 0.134 |  | 0.73 (0.49–1.08) | 0.120 |  | 0.82 (0.57–1.18) | 0.279 |  | 0.76 (0.52–1.12) | 0.164 |
| **Self-rated health** | |  |  |  |  |  |  |  |  |  |  |  |
|  | *Good* | 1.00 (ref) |  |  | 1.00 (ref) |  |  | 1.00 (ref) |  |  | 1.00 (ref) |  |
|  | *Fairly good* | 1.92 (0.97–3.81) | 0.062 |  | 1.64 (1.20–2.26) | 0.002 |  | 2.08 (1.57–2.74) | <0.001 |  | 1.33 (1.00–1.77) | 0.052 |
|  | *Not good* | 3.39 (0.99–11.60) | 0.051 |  | 3.39 (2.18–5.28) | <0.001 |  | 3.94 (2.77–5.60) | <0.001 |  | 3.25 (2.28–4.61) | <0.001 |
| **Satisfaction with life** | |  |  |  |  |  |  |  |  |  |  |  |
|  | *Satisfied* | 1.00 (ref) |  |  | 1.00 (ref) |  |  | 1.00 (ref) |  |  | 1.00 (ref) |  |
|  | *Neither satisfied nor dissatisfied* | 0.45 (0.10–1.97) | 0.290 |  | 0.89 (0.52–1.50) | 0.657 |  | 0.74 (0.48–1.13) | 0.164 |  | 0.86 (0.56–1.31) | 0.477 |
|  | *Dissatisfied* | 2.75 (0.23–33.62) | 0.428 |  | 1.04 (0.49–2.24) | 0.911 |  | 1.05 (0.64–1.74) | 0.838 |  | 1.66 (0.80–3.44) | 0.170 |

**‡** Adjusted odds ratio (OR) and 95% confidence interval (95% CI) with Robust standards errors for use of antibiotics in the previous year. Adjusted for sex, cigarette smoking, deprivation quintiles, self-rated general health, and satisfaction with life.

*a*: *P* value for trend of knowledge score was 0.647 for 16-24 years.

*b*: *P* value for trend of knowledge score was 0.003 for 25-44 years.

*c*: *P* value for trend of knowledge score was 0.017 for 45-64 years.

*d*: *P* value for trend of knowledge score was 0.073 for 65 years or older.

HSNI: Health Survey of Northern Ireland
